# Proteome-Wide Analysis Identifies and Validates Chitinase-3-Like Protein 1 as a Risk and Disease Marker of Delirium

**DOI:** 10.1093/geroni/igaf122.1052

**Published:** 2025-12-31

**Authors:** Sarinnapha Vasunilashorn, Simon Dillon, Tamara Fong, Long Ngo, Hasan Otu, Sharon Inouye, Edward Marcantonio, Towia Libermann

**Affiliations:** Beth Israel Deaconess Medical Center, Boston, Massachusetts, United States; Beth Israel Deaconess Medical Center, Boston, Massachusetts, United States; Beth Israel Deaconess Medical Center, Harvard Medical School, Boston, Massachusetts, United States; Beth Israel Deaconess Medical Center, Boston, Massachusetts, United States; University of Nebraska-Lincoln, Lincoln, Nebraska, United States; Marcus Institute for Aging Research, Hebrew SeniorLife, Harvard Medical School, Boston, Massachusetts, United States; Beth Israel Deaconess Medical Center, Boston, Massachusetts, United States; Beth Israel Deaconess Medical Center, Boston, Massachusetts, United States

## Abstract

Delirium (an acute change in cognition) is a common, morbid, and costly syndrome seen primarily in aging adults. Despite increasing knowledge of its epidemiology, delirium remains a clinical diagnosis with no established biomarkers to guide diagnosis or management. Advances in proteomics now provide opportunities to identify novel markers of risk and disease progression for postoperative delirium and its associated long-term consequences (eg, long-term cognitive decline and Alzheimer’s disease [AD]). In a nested matched case-control study (18 delirium/no-delirium pairs) within the Successful Aging after Elective Surgery study (N = 556), we evaluated the association of 1305 plasma proteins preoperatively [PREOP] and on postoperative day 2 [POD2]) with delirium using SOMAscan. Generalized linear models were applied to enzyme-linked immunosorbant assay (ELISA) validation data of one protein across the full cohort. Multi-protein modeling included delirium biomarkers identified in prior work (C-reactive protein, interleukin-6 [IL6]). We identified chitinase-3-like-protein-1 (CHI3L1/YKL-40) as the sole delirium-associated protein in both a PREOP and a POD2 predictor model, a finding confirmed by ELISA. Multi-protein modeling found high PREOP CHI3L1/YKL-40 and POD2 IL6 increased the risk of delirium (relative risk [95% confidence interval] Quartile [Q]4 vs Q1: 2.4[1.2-5.0] and 2.1[1.1-4.1], respectively). Our identification of CHI3L1/YKL-40 in postoperative delirium parallels reports of CHI3L1/YKL-40 and its association with aging, mortality, and age-related conditions including AD onset and progression. This highlights the type 2 innate immune response, involving CHI3L1/YKL-40, as an underlying mechanism of postoperative delirium, a common, morbid, and costly syndrome that threatens the independence of older adults.

